# Risk Factors for Low Back Disorders in Saskatchewan Farmers: Field-based Exposure Assessment to Build a Foundation for Epidemiological Studies

**DOI:** 10.2196/resprot.5573

**Published:** 2016-06-10

**Authors:** Catherine Trask, Brenna Bath, Peter W Johnson, Kay Teschke

**Affiliations:** ^1^ University of Saskatchewan Saskatoon, SK Canada; ^2^ University of Washington Seattle, WA United States; ^3^ University of British Columbia Vancouver, BC Canada

**Keywords:** back pain, agriculture, occupational exposure, risk factors

## Abstract

**Background:**

Studies of many geographical settings and agricultural commodities show that low back disorders are an important public health issue among farmers, who represent a special rural population. However, few studies have examined the impact of low back disorders on farmers’ work or the strategies that they adopt to avoid associated pain and disability.

**Objective:**

This study protocol will investigate 3 issues related to low back disorders in Saskatchewan farmers: (1) the vibration, heavy lifting, and awkward postures farmers encounter during their work that might contribute to low back disorders; (2) the impact low back disorders have on farmers in terms of their ability to work; and (3) the types of preventative measures and solutions that farmers implement to reduce the occurrence of low back pain.

**Methods:**

To answer these questions, researchers will travel to 30 farms to make measurements of vibration, lifting, and posture during the farmers’ regular work tasks. Farmers will be interviewed about any pain and/or disability using standardized interview questions. Farmers will also be asked about safety measures they have implemented at their farm, such as modified tools or equipment, to reduce the occurrence of low back disorders or pain.

**Results:**

Data collection is currently underway for this study, with the intention to complete all data collection and analysis by the end of 2018.

**Conclusions:**

Occupational determinants of health such as vibration, heavy lifting, and awkward postures are important in the development and progression of low back disorders, and the results of this study will allow for cost-effective epidemiological studies of these determinants in the future. In identifying prevention strategies, this study will also facilitate future research evaluating the effectiveness of safety measures.

## Introduction

Low back disorders are a prevalent and expensive public health problem. Estimates of point prevalence of back pain in the US general population range from 12% to 33%, 1-year prevalence from 22% to 65%, and lifetime prevalence up to 84% [1-3]. In the Netherlands, the estimated total societal cost of back disorder is 1.7% of the country’s gross national product [4]. There is an even greater impact in the agricultural industry; a recent systematic review found that the prevalence of musculoskeletal disorders was consistently higher in farmers than nonfarmer populations, with low back disorders being the most common regional musculoskeletal problem reported [5]. Most surveillance studies have focused on low back disorders, which, as in other industries, represent the bulk of musculoskeletal disorders in farming [5-7]. Across studies of many types of farming, the average lifetime prevalence of low back pain was 75% (95% CI 67-82) and average 1-year prevalence was 48% (95% CI 42-55) [5]. Over the whole working population, low back injuries show particularly high disability, loss of work time, and economic burden [8]. Musculoskeletal disorders have been shown to decrease productivity in construction and industrial workers even when workers do not take time off [9], and farm income is lower when operators have musculoskeletal-related disability [10]. A survey of Iowa farmers showed that they had twice the risk of low back pain compared with the general working population and were 8 times more likely to make major changes in their work activities as a result of low back pain [11].

Reducing the occurrence of low back disorder requires high-resolution, cost-effective exposure assessment techniques to both study disorder mechanisms and identify any changes in exposure that may result from interventions. Epidemiological studies have identified some broad categories of working exposures that are probable risk factors for low back disorders in farmers. Manual material handling or “heavy lifting,” has been shown to be a strong risk factor, with odds ratios ranging from 1.59 to 2.74 [6,12]. Driving tractors has been shown to increase the prevalence of sciatic pain (ie, leg pain associated with low back disorder), unspecified low back pain [13], and the prevalence of lower back and hip joint diseases [12]. This is suspected to be due to whole-body vibration (WBV) and twisted postures during farm vehicle operation, but previous studies have not assessed exposure adequately to determine potential mechanisms. Other categories of work exposure include awkward postures, independent of vehicle operation [6,12,14,15], high work pace and workload [16], and preexisting injury or working with an injury [6]. And, finally, agricultural work tasks have been identified as having high exposure to biomechanical risk factors, although this has yet to be confirmed epidemiologically [17].

However, multiple reviews have cited low-quality exposure assessments as a limiting factor in furthering the understanding of the relationship between farming and work-related musculoskeletal disorders [5,18-21]. Many epidemiological studies characterize exposure via job title or simple self-report, which lack the precision needed to characterize exposure-response relationships, and observation categories such as “low,” “medium,” and “high” are inadequate to detect changes in intervention studies. Some notable exceptions are studies using objective, directly measured exposure, identifying peak and cumulative muscle activity as independent contributors to injury [22], and inclinometer-assessed shoulder posture related to shoulder disorders [23]. Although these objective measures provide a lot of insight, such studies have been rare because of the cost and challenges associated with detailed exposure assessment. When multiple employers or worksites are involved, recruitment and travel can contribute substantially to the cost of on-site electronic data collection [24,25], rendering such measurements impractical for large-scale epidemiological research in the context of traveling to rural farms.

Where direct objective measurements are costly or not feasible, exposure modeling offers an alternative method to extend the utility of direct objective measurements and allow cost-efficient, quantitative exposure assessment for large numbers of workers. Exposure modeling involves concurrent direct objective measurement of exposure and collection of workplace, production, and other characteristics (via observation or surveys) that directly or indirectly increase or decrease physical exposures. The data are used in development of empirical statistical models where characteristics associated with exposure are used to predict exposures in situations where direct measurements cannot be made but where data on the other important work characteristics can be obtained instead. Because surveys are cheaper and allow for multiple measures, the models may provide better estimates of long-term average exposures than direct measurements, which are usually collected for a short time on a small number of individuals. This methodology has long been used in industrial hygiene to estimate a wide variety of airborne exposures [26] such as wood dust [27]. Exposure modeling has also been used successfully for physical exposures, including trunk posture [28], electromyography [29], and WBV [30,31]. An additional benefit of these models is that they identify important determinants of physical exposure and tasks or equipment associated with lower exposures, providing an insight into development of interventions and prevention strategies.

The need for injury prevention research in agriculture has been widely acknowledged [17,32]. Despite this, very few ergonomic interventions have been systematically evaluated in agricultural contexts. Most intervention studies examine exposures with and without modified tools or equipment during lab-based or simulated work [33-35], or in small, uncontrolled field studies [36,37]. Prerequisite for any systematic evaluation of an intervention is an intervention capable of having a substantial effect on adverse working exposures. Existing ergonomic interventions for agriculture (mostly unproven via scientific study) have been primarily targeted toward market fruit and vegetable, nursery, and dairy production [38] rather than large-scale crop farming operations. When identifying workplace controls and interventions, workers are an acknowledged source of information, through both the participatory ergonomics approach [39] and their independent work on equipment modifications and prototypes. It is anticipated that this will be particularly true among farmers given their self-direction and vested interest in productivity; many farmers also have fabrication capacity (ie, skills and equipment for carpentry, welding, and metal fabrication).

This paper describes the design and rationale of a field-based investigation of the risk factors for low back disorders in farmers. This study will quantify the level, duration, and frequency of whole-body (vehicle) vibration, awkward postures, manual material handling, and psychosocial risk factors encountered by farmers at work, then determine whether these exposures can be cost-effectively predicted, evaluated, and modeled using observed and self-reported farm and work task characteristics. Additionally, the study will explore the degree of severity of low back disorder–related pain and disability experienced by farmers, as well as farmer-initiated ergonomic preventative measures, and opportunities for self-initiated prevention of low back disorders identified by farmers.

## Methods

**Figure 1:**
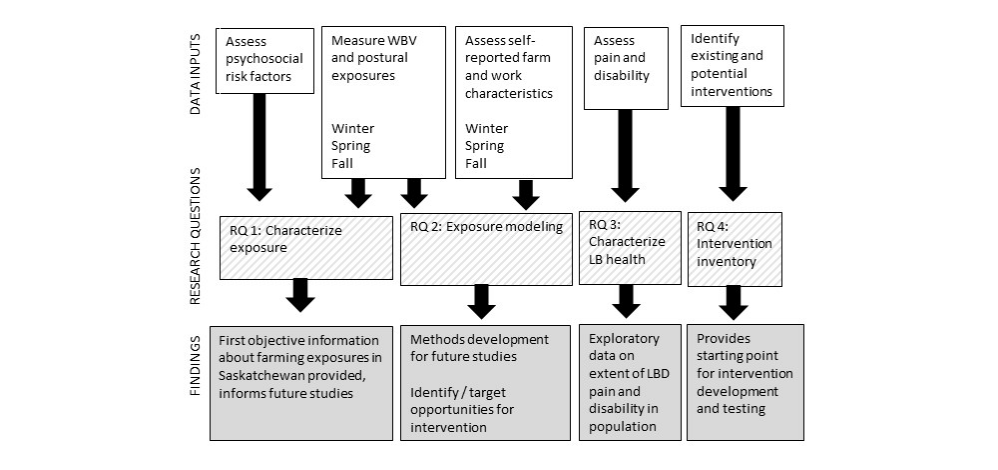
Research design schematic showing the relationship between research questions the data collected in the study (white boxes), the research questions (striped boxes), and contributions to state of knowledge and future research (grey boxes).

To meet its exposure assessment aims, researchers will travel to rural farms throughout the year to collect direct and self-reported exposure data, as well as low back health and information on farmer-initiated interventions. The relationship of collected data to research objectives is shown schematically in Figure 1.

### Study Population, Recruitment, and Sampling Strategy

Farmers in the Canadian province of Saskatchewan are a unique rural population whose low back disorders are largely underserved by both research and occupational prevention efforts. There are 44,329 farms in Saskatchewan, producing commodities like grain, oil seeds, and pulses, as well as poultry, dairy, beef, and pork [40]. These farms require a variety of work tasks: operation of planting and harvesting machinery, equipment and building maintenance, and animal care. The physical demands of these tasks involve exposure to risk factors associated with low back disorders, and farmers have a large cumulative lifetime dose and one of the highest rates of point prevalence for low back pain. Farm work often starts at a very young age and continues beyond typical retirement age [5,41]; 52.7% of Canadian farm operators work more than 40 hours per week on their farm, and 48% also work off the farm [40].

The study will consist of a sample of 24 farms participating in the Saskatchewan Farm Injury Cohort Study. The first phase of this postal survey found that many Saskatchewan farms have mixed production; almost 89% produce grain, 52.7% produce beef, and 6.8% produce other animals [42]. For practical purposes, all eligible farms will reside within 400 km of Saskatoon. Adult principal farm operators will be invited to participate, first by post and then with a follow-up phone call. If farm operators decline to participate, the next randomly selected farm will be invited. In addition to principal farm operators, we will attempt to recruit an additional adult farm worker on each farm for measurement in order to expand the work roles and task types included in the study. Additional workers will be eligible if they perform farm tasks at least 12 weeks of the year. Given the occupational structure indicated by prior surveys of this group, we anticipate being able to assess 2 people on at least 50% of farms [42] for a total of 36 participants in the study. Each farm will be visited for on-site data collection 3 times during a calendar year for a total of 108 farm measurements. Measurements will be scheduled throughout a 1-year period to account for seasonal variability in work tasks and exposures: spring (planting, April-June), fall (harvest, August-September), winter (land and equipment maintenance, November-March).

### Data Collection

Three types of data collection will be performed during measurement visits: direct measurement of exposure using direct objective measurements of physical exposures; a structured in-person interview on back health and occupational exposures; and a semistructured interview to identify any ergonomic or safety-related measures designed to reduce or mitigate exposure to low back risk factors. This data collection will be quite intensive. The recruitment, scheduling, and travel involved in on-farm data collection will be substantial but will provide an opportunity to collect information that will support hypothesis generation and evaluate measurement strategies for feasibility. Measurement protocols will be developed, pilot-tested, and refined with the collaboration of the Prairie Agricultural Machinery Institute (PAMI) and University of Saskatchewan research farms; this will ensure that the methods are feasible in farming contexts and minimize disruption of work activities.

Direct objective measurements of WBV, trunk posture, and manual material handling and a brief exposure self-report will be made on all 3 seasonal farm visits for the duration of farm work activities. Mounting, calibrating, and removing equipment will take approximately 20 minutes. Measurement visits will be scheduled by phone to determine a start time and the anticipated duration of measurements.

#### Direct Exposure Measurements

Whole-body vibration from vehicles will be measured according to the ISO 2631 guidelines [43] with triaxial accelerometer (Series 2A triaxial accelerometer, NexGen Ergonomics, Montreal, Canada) placed at the seat-operator interface. Vibration data will be recorded using a MWX8 DataLOG (Biometrics Ltd, Newport, UK) with a sampling rate of 1000 Hz, then analyzed using custom software to determine standard exposure metrics including time-weighted 8-hour root-mean-square (RMS) A(8); 8-hour vibration dose value, VDV(8); and 8-hour static compressive dose, Sed(8).

Trunk posture will be quantified using wearable, data-logging, battery-powered inertial sensors (I2M Inertial Measurement Unit, Human Motion Analysis), which allow for dynamic assessment and high-resolution data logging in 3 dimensions: flexion-extension, lateral flexion, and spinal rotation. These data will be analyzed using Human Motion Analysis software to develop common research exposure metrics: amplitude probability distribution function percentiles (10th, 50th, and 90th), rest periods, and percentage of time spent above key exposure levels: >15, >30, > 45, >60, and 90 degrees of trunk flexion [44].

#### Observation of Manual Handling

Manual material handling tasks, including vehicle and building maintenance, animal care, and seed preparation, will be video-recorded using a digital video camcorder (Sony Handycam HD). Exposure to manual handling will be summarized by reviewing the data with Observer XT event-logging software (Noldus Information Technology Inc, Leesburg, USA) into daily counts and durations, and then compared with risk assessment guidelines such as the National Institute for Occupational Safety and Health (NIOSH) lifting equation, American Conference of Industrial Hygienists (ACGIH) Lifting threshold limit value (TLV), University of Michigan’s Three-Dimensional Static Strength Prediction Program (3DSSPP) and psychophysical acceptability tables [45,46].

#### In-Depth Interview on Exposure and Low Back Health

A structured, in-person, on-farm interview will be conducted once during the winter visit, at periods that will not be disruptive to workflow. The interview will include sections on working exposures to risk factors associated with low back disorders and adverse low back health. Farm, vehicle, and task characteristics potentially related to exposures (see Table 1) will be assessed using previously published questionnaire items, where available [28,30,47,48]. Exposure questions will assess “typical” exposures and the variability of exposures throughout the year.

**Table 1:** Directly measured exposure categories and potential determinants of exposure.

Exposure	Proposed measurement sample	Potential determinants of exposure
Whole-body vibration	21 farms × 6 vehicles each=126 vehicle measurements (anticipate 2 vehicles per visit)	Vehicle characteristics: type of vehicle or other vibrating equipment used, operating duration, vehicle weight, type of tire, type of transmission, seat type, seat and cab suspension. Driving surface characteristics. Driving tasks: duration of operation, typical speeds of operation.
Back posture	36 farmers × 3 days=108 posture measurements	Duration and frequency of farm tasks such as shoveling, vehicle maintenance, animal feeding and watering, birth and veterinary care, vehicle operation, and other tasks identified during the pilot phase. Horizontal reach distances, frequency and extent of bending or twisting and reaching overhead.
Manual handling	36 farmers × 3 days=108 hours of video (anticipate 1 hour of manual tasks per visit)	Duration and frequency of farm tasks such as shoveling, vehicle maintenance, animal feeding and watering, birth and veterinary care, vehicle operation, and other tasks identified during the pilot phase. Dimensions and estimated weights of materials handled, the heights over which they are transported, the use of lifting aids, horizontal distance of load from ankles, vertical distance of load from floor, movement distance, amount of twisting, lift frequency and duration, presence of handles.

Farmers will also be asked about psychosocial risk factors using Karasek’s Job Content Questionnaire [49]. The low back health portion of the interview will use existing, validated instruments to collect in-depth information on the presence and extent of pain, and any interruption of work, family, leisure, and activities of daily living [50-53]. As part of inclinometer assessment of back posture, spine range of motion will be directly measured. These data will be used to characterize back structure and function. The in-depth, on-farm interview and assessment is anticipated to take 60-75 minutes. Items will use terms familiar to the workforce, refined during consultations with industry partners and during pilot testing. Images of key postures, tasks, and lifting activities will be offered to assist recall.

#### Developing an Intervention Inventory

An inventory of farmer-initiated interventions will be assembled using targeted questions during the in-depth, on-farm interview, anticipated to take 10-45 minutes depending on the number of safety measures in place. The focus will be on engineering interventions that involve physical changes to tools, equipment, machinery, or workstations, but administrative interventions such as work rest and micropause schedules will also be considered, as well as self-care strategies. Engineering interventions are preferred because they have been shown to have the greatest economic benefit [54] and, in contrast to administrative controls, are not as reliant on the farmer to remember to take action. Qualifying interventions may be modifications currently or previously used by the farmer, repurposed or custom-built tools or equipment, interventions that are in the planning or fabrication stage, as well as interventions that have only been identified as a need but not yet implemented. When an intervention is identified, additional information will be collected to determine the nature and utility of the intervention; for example, what prompted implementation; how the device/practice was acquired or developed; strengths and limitations of the device/practice; satisfaction with the device/practice; and a “wish list” of any improvements to the device/practice. When possible, photographs of a device and video of its use will be collected and will be accessible on the project website. During pilot testing, a semistructured interview will be used to collect qualitative data on these topics. It is anticipated that this process will allow identification of critical intervention characteristics (low, medium, and high cost; short, medium, and long time frame for implementation/adoption) to provide more structure during study data collection.

### Analysis

#### Direct Exposure Measurements

Analysis of these exposure data will include descriptive summaries for all measurement days combined as well as for each commodity, season, and other categories. Differences between categories will be assessed using mixed models with “farmer” and “farm” as random-effects terms. Repeated measures from all 3 visits will be used to estimate within-worker variability and compare it with between-worker variability, which can be used to develop cost-efficient sampling strategies as suggested by Burdorf and Van Riel [55]. Self-reported “typical” exposure and “annual variability” will be compared with direct measurements using linear regression modeling to determine the suitability of “overall” exposure estimates to represent daily exposures that are anticipated to be highly variable.

#### Exposure Prediction Modeling

All potential prediction variables will be summarized descriptively and tested for association with measured exposures using simple linear regression. Some example variables are listed in Table 1. As done in previous work, variables with a *P* value of less than .10 will be retained for further consideration [28-30]. Colinearity will be assessed before offering to the multiple regression model. Interactions will be considered where there is a theoretical basis or published evidence. Similar to prior work [28,56], hierarchical multiple linear regression models (ie, mixed-effects models) will be developed using “farmer” and “farm” as random-effects terms and potential predictor variables as fixed effects. Manual stepwise backward regression will be conducted and significant variables will be retained in the final model.

#### Intervention Inventory

Identified interventions will be cataloged into an inventory according to their application (ie, relevant commodities, tasks, and equipment). After a heuristic review by trained ergonomists (Drs PJ and CT), the cataloged interventions will be published on the project website. Interventions characteristics such as cost, time commitment, and associated barriers and facilitators will be summarized into frequencies. The NIOSH agricultural best practices guide [38] and International Labour Organization’s Ergonomics Checkpoints guide [57] will provide a model for an intervention inventory, tailored to farming conditions encountered in Saskatchewan.

#### Sample Size

With regard to statistical power, Mathiassen et al [58] demonstrated that in relatively constrained industrial work, the number of subjects needed to detect significant differences depended on the sizes of the within- and between-subject variability components. In order to detect a 10% difference with 80% power, 15 subjects are required when measuring joint angle (using posture sensors as proposed in this study). Given that the work tasks involved in Saskatchewan farming are much less constrained than in most industrial settings, considerable heterogeneity in work exposures is expected, therefore 36 individuals with 3 repeated measurements are expected to provide an adequate sample size. The number of measurements (~36 subjects times 3 days=~108) is likely to limit the number of potential determinants in each empirical model of exposure. However, previous determinants of exposure studies required only 4 variables to predict posture [28] and 3 variables to predict vehicle vibration [30]. The proposed research anticipates including no more than 7 variables in the exposure prediction models, which should be well supported by the proposed sample size [59].

Because the primary research aims do not test typical exposure-response hypotheses, we cannot perform power calculations. We acknowledge that sample size will not make a representative sample of low back disorder prevalence or implementation of ergonomic interventions; however, these are preliminary explorations of this area intended to generate hypotheses and evaluate the feasibility of the methods for further study.

#### Ethics

The biggest potential burden for participants will be the amount of time required to set up and remove measurement equipment during busy workdays (approximately 15-25 minutes in total). Data collectors will thoroughly practice equipment preparation to mitigate this. The longer in-person interview (60-75 minutes in total) will be scheduled during the less busy winter season to minimize disruption of work activities. The study protocol and consent forms have been approved by the University of Saskatchewan Behavioural Research Ethics Board.

## Results

Data collection is currently underway for this study, with the intention to complete all data collection and analysis by the end of 2018.

## Discussion

### Research Benefits: a Foundation for Future Studies

Outcomes of the proposed study will have value by providing methods for future research and immediate applicability to farmers and agricultural health and safety organizations. This project will provide objective, high-quality, directly measured exposure information in the understudied population of rural famers. Providing high-quality data on the physical exposures in agriculture allows for comparison with exposures in other industries and occupations and, where available, exposure guidelines. Such comprehensive measurements of these determinants of health status are rare in agriculture and have not been conducted in Saskatchewan, where farm and agriculture revenue makes substantial contributions to the local economy. This study will also provide estimates of the extent and nature of low back disorder–related pain and disability in farmers; by better characterizing the scope of the problem of low back disorder it will provide rich information on low back disorders’ effect on work and home life and back function.

The study will also contribute to future research by providing descriptive data on exposures and low back disorder health that will support development of hypotheses, methodologies, and designs for future research. In terms of epidemiology, developing statistical modeling techniques for cost-effective exposure assessment will allow for higher-quality, lower-cost, large-scale studies in the future. Poor exposure assessment is a persistent problem in prevention of low back disorders. A major outcome of this project will be the development of a parsimonious, cost-effective exposure prediction model to identify determinants of back health status during large, prospective epidemiological studies. By developing an exposure prediction model, the proposed study will determine if farmers can report farm tasks and working conditions accurately enough to develop empirical models of WBV, back posture, and manual material handling to make quantitative predictions of working exposures. For example, this model could identify a simple set of questions that are demonstrably related to exposure for a baseline exposure assessment via postal questionnaire. This would be an advance over current self-reported exposure questionnaires that have face validity but without a quantified relationship between reported and measured exposures. It should be noted that this type of model develops a relationship based on exposures measured on a specific day. However, many musculoskeletal disorders, including low back disorders, develop from long-term exposures accumulated over time. Therefore, models that rely on predictors that vary over time may be less representative on relevant long-term exposures than those predictors that are relatively fixed.

Many of the applications of the findings could be translated to other industries. For example, mining is one industry where many of the same exposures (WBV and lifting) exist. In addition, identifying the determinants of exposure will allow for better targeting of prevention efforts, because these workplace factors can be modified or controlled.

Perhaps most relevant to immediately applicable prevention efforts will be the creation of an ergonomic intervention inventory describing existing and potential farmer-initiated preventative measures. This will provide a great deal of quantitative and qualitative information to support future prevention research. In addition to identifying promising designs and strategies for exposure control, the inventory can provide insight into why these interventions are implemented, including barriers and facilitators that can predict the success of future intervention designs; this will be published on the study website. This inventory will facilitate identification of promising designs, strategies, and opportunities for future research that investigates effectiveness of interventions.

### Relevance

This project directly addresses an important health issue in an understudied population with high anticipated risks for the development of low back disorders. It represents the first phase in a longer-term research program to investigate the etiology of back disorders in this group. The results of this research will ultimately provide evidence to inform policy and prevention program decisions.

## Abbreviations

ISO: International Organization for Standardization
NIOSH: National Institute for Occupational Safety and Health
WBV: whole-body vibration
